# Carotid endarterectomy and the risk of perioperative stroke: The importance of chronic ischaemic lesions and small vessel disease

**DOI:** 10.1111/ene.16551

**Published:** 2025-01-03

**Authors:** Henrietta Törmänen, Suvi Koskinen, Krista Nuotio, Pirkka Vikatmaa, Petri T. Kovanen, Lauri Soinne, Perttu J. Lindsberg, Petra Ijäs

**Affiliations:** ^1^ Neurology University of Helsinki and Helsinki University Hospital Helsinki Finland; ^2^ Clinical Neurosciences, Clinicum University of Helsinki Helsinki Finland; ^3^ Department of Radiology Hospital District of Helsinki and Uusimaa Medical Imaging Center, University of Helsinki and Helsinki University Hospital Helsinki Finland; ^4^ Department of Vascular Surgery University of Helsinki and Helsinki University Hospital Helsinki Finland; ^5^ Wihuri Research Institute Helsinki Finland

**Keywords:** carotid stenosis, perioperative stroke, small vessel disease

## Abstract

**Background and purpose:**

Perioperative stroke is a well‐recognized complication of carotid endarterectomy (CEA), but well‐performing prediction models do not exist for it. Our aim was to identify novel predictors for perioperative ischaemic cerebrovascular events (iCVEs), emphasizing cerebrovascular imaging and potential biomarkers for stroke in carotid stenosis (CS) patients in a well‐characterized prospective CS cohort.

**Methods:**

Helsinki Carotid Endarterectomy Study 2 is an observational prospective and consecutive cohort study of CS patients subjected to CEA during 2012–2015. The associations between perioperative stroke and transient ischaemic attack (iCVEs) and potential predictive factors were evaluated by univariate and Cox regression analyses.

**Results:**

Of 488 operated CS patients, 33 (7%) sustained an iCVE including 21 (4%) ischaemic strokes. In univariate analysis, moderate ipsilateral CS (hazard ratio [HR] 2.14, 95% confidence interval [CI] 1.08–4.23), covert or chronic ipsilateral brain infarct in imaging (HR 2.27, 95% CI 1.09–4.76) and severe cerebral small vessel disease (HR 3.36, 95% CI 1.04–10.88) appeared as novel risk factors for perioperative iCVE. In Cox proportional hazards regression modelling, female gender (HR 3.03, 95% CI 1.30–7.04), a history of coronary heart disease (HR 3.59, 95% CI 1.52–8.47), covert or chronic ipsilateral infarct (HR 2.32, 95% CI 1.01–5.34) and severe small vessel disease (HR 2.63, 95% CI 1.07–6.47) were the strongest independent predictors of perioperative iCVE.

**Conclusions:**

In addition to the previously reported clinical risk factors, it was found that imaging markers of past cerebrovascular disease, covert or chronic ipsilateral infarct and severe small vessel disease, and moderate ipsilateral stenosis are associated with perioperative iCVEs.

## INTRODUCTION

Carotid endarterectomy (CEA) is the gold standard for reducing the risk of stroke in symptomatic and selected asymptomatic severe carotid stenosis (CS) patients. CEA is more effective than medical therapy based on large, randomized trials [1] in experienced treatment centres where the rate of any periprocedural (30‐day) stroke or death is low, less than 6% for symptomatic and 3% for asymptomatic patients. The risk of perioperative stroke varies from 1.4% to 5.1% in different trials, depending on patient characteristics, timing of surgery and experience of the surgeon [2, 3, 4, 5, 6, 7].

The pathophysiology behind perioperative stroke after CEA is miscellaneous. Intraoperative or postoperative embolization from luminal thrombi as well as intraoperative or postoperative CEA hypoperfusion or hyperperfusion are the most common mechanisms. However, the exact cause of perioperative stroke may be difficult to establish. Bleeding complications exist as perils after CEA, with intracerebral haemorrhage (ICH) associated with cerebral hyperperfusion syndrome being the most feared [8]. Neck haematoma occurs in 0.6% to 15.8% of postoperative complications and may necessitate reoperation, which in turn fuels increased risk of in‐hospital myocardial infarction (MI), stroke or death [9], but may be reduced with intraoperative administration of protamine [10, 11, 12].

Randomized clinical trials [11, 12, 13], large registries or healthcare databases [14, 15] as well as single centre/cohort studies [16] have reported clinical and radiological risk factors for perioperative stroke. Well‐defined risk factors include age, female gender, coronary heart disease (CHD), type of presenting symptom (ischaemic stroke or crescendo transient ischaemic attack [TIA] compared to amaurosis fugax [AFX]), left‐sided CEA, high preoperative blood pressure, contralateral stenosis or occlusion, and acute ipsilateral infarct in computerized tomography/magnetic resonance imaging (CT/MRI) [17, 18, 19]. Prediction models have also been developed but their discrimination has been shown to be poor [17]. Most studies have concentrated on risk factors readily available in register‐based studies, such as demographics and comorbidities, that may not reveal relevant information on a high‐risk unstable carotid plaque or compromised cerebrovascular regulation, both typical for high‐risk CS patients.

The Helsinki Carotid Endarterectomy Study 2 (HeCES2) is a longitudinal prospective cohort study that enrolled 500 consecutive CS patients scheduled for CEA in 2012–2015. Detailed clinical, radiological (brain and carotid imaging) and biomarker data were collected on the patients. The aim of this study was to search for novel risk factors for perioperative ischaemic cerebrovascular events (iCVEs) with an emphasis on cerebrovascular imaging and potential biomarkers for stroke and unstable CS.

## METHODS

The HeCES2 is a cross‐sectional and longitudinal prospective cohort study that was conducted at the Helsinki University Hospital in Helsinki, Finland, in collaboration with the Departments of Neurology, Vascular Surgery and Radiology. The HeCES2 study recruited 500 consecutive symptomatic or asymptomatic CS patients scheduled for CEA from October 2012 until September 2015. In all, 488 patients underwent CEA, of whom 37 were operated on bilaterally. A detailed description of the study protocol can be found elsewhere [18]. The Medical Ethics Committee of Helsinki and Uusimaa District approved the HeCES2 study, and all study patients agreed to participate and gave their written informed consent.

A patient was classified as symptomatic if she/he had suffered an ipsilateral AFX, TIA or ischaemic stroke within 180 days prior to CEA. The presenting event was defined as the key ischaemic cerebrovascular symptom that led the patient to seek medical assistance. The event was registered according to the most severe symptom; for example, if the patient suffered from a hemispheric TIA and AFX, the hemispheric TIA was registered as the presenting symptom. Patients whose symptoms could not be determined if they were derived from the CS or possible other aetiology were classified as ‘suspect’.

A vascular surgeon or neurologist initiated the best medical treatment according to the institutional and national guidelines of that time (Table 1) [19, 20, 21]. Of all patients 51% (251) were on single‐antiplatelet therapy, 4.5% (22) were on dual‐antiplatelet therapy (DAPT) and 56% (273) were on some form of low molecular weight heparin most commonly as a bridging therapy due to pre‐existing anticoagulation medication.

**TABLE 1:** Baseline characteristics in the carotid stenosis patients with and without any perioperative ischaemic cerebrovascular event (AFX, TIA, stroke, symptom worsening).

Variable	All CEA patients *n* = 488	Patients without iCVE *n* = 455 (93%)	Patients with iCVE *n* = 33 (7%)	Univariate *p* *	Age‐adjusted HR for iCVE	Age‐adjusted HR for any TE
Gender (male)	330 (67.6)	310 (68.1)	20 (60.6)	0.441	0.78 (0.38–1.58)	0.95 (0.50–1.79)
Age (mean/SD)	69.6 ± 8.5	69.6 ± 8.4	71.6 ± 9.3	0.208		
Smoking	178 (36.5)	168 (36.9)	10 (30.3)	0.464	0.90 (0.39–2.08)	1.19 (0.58–2.46)
Heavy alcohol consumption	52 (10.7)	50 (11.0)	2 (6.1)	0.418	0.63 (0.15–2.69)	0.47 (0.11–1.96)
CEA side (left %)	235 (48.2)	214 (47.0)	21 (63.6)	0.048	1.89 (0.91–3.79)	1.42 (0.77–2.61)
Type of the presenting event						
Stroke	136 (27.9)	122 (26.8)	14 (42.4)	0.069	1.20 (0.57–2.53)	1.26 (0.65–2.44)
Ocular stroke (RAO)	24 (4.9)	23 (5.1)	1 (3.0)	0.722	0.46 (0.06–3.52)	0.37 (0.05–2.75)
AFX	82 (16.8)	81 (17.8)	1 (3.0)	0.028	0.15 (0.02–1.11)	0.24 (0.06–1.04)
TIA	79 (16.2)	76 (16.7)	3 (9.1)	0.332	0.44 (0.13–1.53)	0.59 (0.22–1.60)
*N* of symptoms^a^	1 (1–6.5)	2 (1–6)	4.5 (1–20.75)	0.624	0.69 (0.25–1.90)	1.00 (0.81–1.23)
Suspect	67 (13.7)	63 (13.4)	4 (12.1)	1.000	0.79 (0.28–2.27)	1.19 (0.51–2.75)
Asymptomatic	100 (20.5)	90 (19.3)	10 (30.3)	0.178	1.92 (0.90–4.12)	1.41 (0.67–2.96)
Comorbidities						
Hypercholesterolaemia	446 (91.4)	414 (91.0)	32 (97.0)	0.344	3.04 (0.42–22.3)	0.84 (0.25–2.84)
Hypertension	397 (81.4)	369 (81.1)	28 (84.8)	0.655	1.08 (0.41–2.84)	0.86 (0.40–1.92)
Diabetes	163 (33.4)	151 (33.2)	12 (36.4)	0.849	1.04 (0.50–2.13)	0.96 (0.50–1.83)
CHD	181 (37.1)	161 (35.4)	20 (60.6)	0.005	2.44 (1.18–5.05)	2.72 (1.45–5.10)
AF	84 (17.2)	76 (16.7)	8 (24.2)	0.336	1.23 (0.54–2.83)	131 (0.60–2.84)
Medications						
SAPT	251 (51)	236 (52)	15 (45.5)	0.589	0.81 (0.41–1.61)	1.40 (0.59–3.33)
DAPT	22 (4.5)	20 (4.4)	2 (6.1)	0.654	1.69 (0.38–7.57)	1.44 (0.27–7.79)
LMWH	273 (55.9)	252 (55.4)	21 (63.6)	0.372	1.37 (0.64–2.92)	2.04 (0.81–5.14)
Acute recanalization therapies						
IVT	23 (4.7)	22 (4.8)	1 (3.0)	1.000	0.65 (0.09–4.77)	1.03 (0.25–4.24)
EVT	1 (0.2)	1 (0.2)	0	1.000	ND	ND
Clinical measures						
SBP at arrival^b^	153 ± 26.8	152 ± 26.9	162 ± 22.5	0.086	1.01 (1.00–1.03)	1.01 (0.97–1.03)
DBP at arrival^b^	79 ± 15.2	78 ± 14.9	83 ± 19.2	0.153	1.02 (1.00–1.05)	1.01 (0.98–1.03)
Time to CEA^c^	11 (7–19)	11 (7–21)	10 (4–15)	0.128	0.96 (0.91–1.01)	0.99 (0.98–1.09)

*Note*: Data are presented as *n* (%), mean ± standard deviation or median (interquartile range). Chi‐squared or Fisher's exact test for dichotomous or categorical variables, Mann–Whitney *U* test for ordinal variables and Student's *t* test for continuous variables were used. In the left‐sided CEA group are all left‐sided CEAs and the bilateral CEAs where the left side was treated first. Patients who still smoked for as long as 1 year before the index symptom were classified as smokers. Heavy alcohol consumption in women was rated as more than 12–16 doses (one dosage of alcohol is 12 g of pure ethanol) and in men more than 23–24 doses of alcohol per week.

Abbreviations: AF, atrial fibrillation; AFX, amaurosis fugax; CEA, carotid endarterectomy; CHD, coronary heart disease; DAPT, dual‐antiplatelet treatment; DBP, diastolic blood pressure; EVT, endovascular thrombectomy; HR, hazard ratio; iCVE, ischaemic cerebrovascular event; IVT, intravenous thrombolysis; LMWH, low molecular weight heparin; RAO, retinal artery occlusion; SAPT, single‐antiplatelet treatment; SBP, systolic blood pressure; TE, thromboembolic event; TIA, transient ischaemic attack.

* *p* < 0.05 is considered statistically significant.

^a^ For TIA patients.

^b^ Preoperative blood pressure values available for 375 patients.

^c^ For symptomatic patients.

Prior to surgery, the patients underwent brain CT or MRI, examination of carotid arteries with duplex ultrasound and CT or MR angiography. The degree of CS (%) was calculated using the North American Symptomatic Carotid Endarterectomy Trial (NASCET) criteria [18, 22]. The cutoff for a significant stenosis was defined as 50% or over. The over 1–2‐mm‐deep ulcerations visible in CT axial images as contrast‐filled ‘pouches’ were scored as ‘ulcerations’. Ischaemic parenchymal CT/MRI changes were classified according to the age and region of the lesion. As the pragmatic cutoff for near‐occlusion, a 1.0‐mm side‐to‐side difference in distal internal carotid artery luminal diameter was utilized. Covert infarctions were defined as focal lesions detected on brain imaging for which brain ischaemia seems the most likely explanation, but one in which the patients lacked a congruent history of a clearly attributable acute neurological dysfunction [23]. Fazekas classification was used to grade the subcortical white matter lesions (cerebral small vessel disease) [24]. Severe leukoaraiosis was defined as Fazekas classification 3.

Blood samples were drawn within 2 weeks perioperatively. Laboratory investigations that have been shown to be associated with a risk of ischaemic stroke in CS or general ischaemic stroke patients were investigated [25, 26]. High‐sensitivity C‐reactive protein, fibrinogen, interleukin 6, apolipoprotein B/apolipoprotein A ratio, low‐density lipoprotein cholesterol and low density/high density lipoprotein cholesterol ratio were analysed (Table 2).

**TABLE 2:** Laboratory parameters in the carotid stenosis patients with and without any perioperative ischaemic cerebrovascular event (AFX, TIA, stroke, symptom worsening).

Variable	All CEA patients *n* = 488	Patients without iCVE *n* = 455 (93%)	Patients with iCVE *n* = 33 (7%)	Univariate *p* *	Age‐adjusted HR for iCVE	Age‐adjusted HR for any TE
Hs‐CRP	5.3 ± 11.9	5.2 ± 11.0	8.2 ± 0.7	0.170	1.01 (0.99–1.03)	1.01 (1.00–1.03)
Fibrinogen	4.0 ± 0.2	4.0 ± 0.9	3.8 ± 0.8	0.415	0.88 (0.58–1.32)	0.94 (0.66–1.34)
ApoB/ApoA ratio	0.6 ± 0,3	0.6 ± 0.3	0.7 ± 0.4	0.136	2.26 (0.94–5.48)	1.66 (0.88–3.10)
LDL‐cholesterol	2.5 ± 1.0	2.5 ± 1.0	2.2 ± 0.9	0.116	0.99 (0.68–1.45)	0.78 (0.45–1.38)
LDL/HDL cholesterol ratio	2.0 ± 0.9	2.0 ± 0.9	2.0 ± 1.0	0.828	0.84 (0.51–1.37)	1.03 (0.57–1.86)
IL‐6	53 ± 233	56 ± 241	13 ± 22	0.309	0.99 (0.99–1.02)	1.00 (0.99–1.01)

*Note*: Data are presented as *n* (%), mean ± standard deviation or median (interquartile range).

Abbreviations: ApoA, apolipoprotein A; ApoB, apolipoprotein B; CEA, carotid endarterectomy; HDL, high‐density lipoprotein; Hs‐CRP, high‐sensitivity C‐reactive protein; iCVE, ischaemic cerebrovascular event; IL‐6, interleukin 6 (pg mL–^1^); LDL, low‐density lipoprotein; TE, thromboembolic event; TIA, transient ischaemic attack.

* *p* < 0.05 is considered statistically significant.

All patients had patch angioplasty, mostly a polyester‐urethane patch. All were operated either under general or local anaesthesia and both transcranial Doppler (TCD) and near‐infrared spectroscopy (NIRS) monitoring were used during the active period. Shunting was performed selectively based on the surgeon's evaluation of the findings in TCD and/or NIRS or symptoms in the awake patient. When the flow was less than 100 mL/min routine, completion control was achieved, using transit time flow measurement and ultrasound to evaluate problems.

An iCVE was defined as patients who had a perioperative ischaemic stroke or TIA or worsening of symptoms. In patients where it was not clear whether the neurological symptoms were TIA or worsening of earlier symptoms were classified as TIA/worsening of symptoms.

Patients with perioperative iCVEs were divided into three groups. In the first group all who suffered from an iCVE during the operation or up to 6 h beyond it were included. In the second group were all who experienced an iCVE during their hospital stay, and in the third group all patients who experienced a perioperative iCVE after hospital discharge within 30 days of CEA. A larger group, ‘the thrombotic event group’ included all the patients who suffered from a perioperative iCVE (within 6 h of CEA, during hospital stay or after hospitalization) and those who experienced an MI/angina pectoris during the 30‐day follow‐up.

A perioperative stroke was described as a new neurological deficit lasting longer than 24 h during the 30 days after CEA. A stroke resulting in a decline of more than 2 points in the perioperative modified Rankin Scale (mRS) was considered a disabling stroke (major stroke), all others as non‐disabling strokes (minor strokes).

Bleeding complications were divided into three categories. In the first category was ICH due to hyperperfusion syndrome. In the second category were neck haematomas requiring evacuation. In the third category were all the patients who had prolonged haemostasis; this included all patients who had a comment on prolonged haemostasis during the surgery in the medical report, had large volumes of wound drainage postoperatively (over 500 mL) or an emergency department visit in which a neck haematoma and/or bleeding were established.

The patients' demographic data are given as mean and standard deviation for normally distributed variables, or median and interquartile range. Univariable and multivariable analyses were used in the statistical analyses of potential predictors for iCVEs and thrombotic events. Chi‐squared or Fisher's exact test was used for dichotomous variables, Student's *t* test for normally distributed continuous variables and the Mann–Whitney *U* test for categorical variables and non‐normally distributed continuous variables. The age‐adjusted hazard ratios of potential predictors for iCVEs were calculated by Cox binary logistic regression analysis. Cox proportional hazards regression modelling was used to investigate the association between the iCVE‐free survival time and potential predictor variables. Time was expressed in days to either iCVE or the end of the 30‐day follow‐up. Two multivariable models were created: (i) a model including age, sex and the variables significant by univariate analysis, and (ii) a model with age, sex and the variables showing association with a *p* value below 0.20 in the univariate analysis but subjected to backward elimination. IBM SPSS Statistics 22 software was used for statistical analyses.

## RESULTS

**FIGURE 1:**
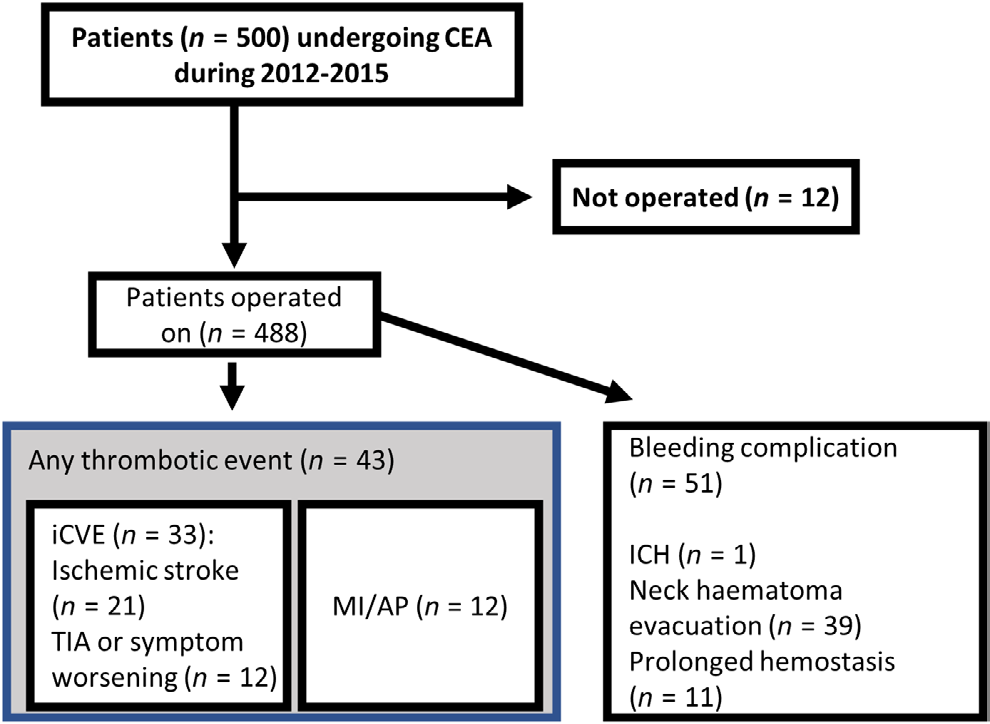
Flowchart of the study. Reasons for withdrawing the decision for CEA: recurrent disabling stroke (*n* = 1), progression to total carotid occlusion (*n* = 2), no significant stenosis (*n* = 3), carotid aneurysm filled with thrombus (*n* = 1), thrombectomy (*n* = 1), dilation operation (*n* = 1), symptoms were not derived from carotid artery stenosis (*n* = 1), stenosis at carotid siphon combined with pre‐stenotic collapse (*n* = 1) and general weakness (*n* = 1). AP, angina pectoris; CEA, carotid endarterectomy; ICH, intracerebral haemorrhage; iCVE, ischaemic cerebrovascular event; MI, myocardial infarction; TIA, transient ischaemic attack.

Five hundred symptomatic or asymptomatic CS patients scheduled for CEA were recruited in the HeCES2 study. In all, 488 patients underwent CEA for their CS (Figure 1); 321 (66%) patients were symptomatic, 100 (20%) patients asymptomatic and 67 (14%) patients' symptoms were classified as ‘suspect’. Of all patients 380 had a head CT and 62 had an MRI; 46 patients did not have any kind of scanning of the brain. The mean age was 69.6 (±8.5) years, and most of the patients were men (68%). Twenty‐three (4.7%) got intravenous thrombolysis (IVT) when admitted to the hospital. Detailed clinical characteristics of the patients are shown in Table 1.

The reasons given for withdrawing the decision for CEA (*n* = 12, Figure 1) were recurrent disabling stroke (*n* = 1), progression to total carotid occlusion (*n* = 2), no significant stenosis after the neuroradiologist's review (*n* = 3), carotid aneurysm filled with thrombus (*n* = 1), thrombectomy resulting from no plaque (*n* = 1), dilation operation (*n* = 1), symptoms not derived from CS (*n* = 1), stenosis at carotid siphon combined with pre‐stenotic collapse (*n* = 1) and general weakness (*n* = 1).

Of the 488 patients who underwent CEA, 33 (7%) experienced an unexpected perioperative iCVE (including ischaemic stroke, TIA or worsening of symptoms) (Table 3). Twenty‐one patients (4%) incurred an ischaemic stroke and 12 (2%) suffered from a TIA or worsening of symptoms. None had AFX. Of all the 33 perioperative iCVEs, 16 (48%) occurred within 6 h of CEA, another 14 (42%) during later hospitalization and finally three (9%) after hospitalization within 30 days. One patient (0.2%) had an ICH due to cerebral hyperperfusion syndrome. Thirty‐eight patients (7.8%) had to undergo evacuation of a neck haematoma after CEA, whilst an additional 11 (2.3%) developed prolonged haemostasis. Twelve patients (2.5%) suffered from an MI or angina pectoris. A detailed description of the perioperative complications is shown in Table 3.

**TABLE 3:** Perioperative complications in carotid stenosis patients undergoing CEA.

Perioperative complication	All CEA patients (*n* = 488)	CEA patients by the presenting symptom					
		All symptomatic (*n* = 321)	Ischaemic stroke or RAO (*n* = 160)	TIA (*n* = 79)	AFX (*n* = 82)	Suspect (*n* = 67)	Asymptomatic (*n* = 100)
Ischaemic stroke, TIA or symptom worsening	33 (6.8)	19 (5.9)	15 (9.4)	3 (3.8)	1 (1.2)	4 (6.0)	10 (10)
Within 6 h of CEA	16 (3.3)	10 (3.1)	9 (5.6)	0	1 (1.2)	2 (3.0)	4 (4.0)
During hospital stay	14 (2.9)	8 (2.5)	6 (3.8)	2 (2.5)	0	1 (1.5)	5 (5.0)
After hospitalization, within 30 days	3 (0.6)	1 (0.3)	0	1 (1.3)	0	1 (1.5)	1 (1.0)
Ischaemic stroke	21 (4.3)	13 (4.0)	12 (7.5)	1 (1.3)	0	2 (3.0)	6 (6.0)
Disabling stroke (mRS >2)	8 (1.6)	6 (1.9)	6 (3.8)	0	0	0	2 (2.0)
Non‐disabling stroke (mRS 0–2)	13 (2.7)	7 (2.2)	6 (3.8)	1 (1.3)	0	2 (3.0)	4 (4.0)
TIA or symptom worsening	12 (2.5)	6 (1.9)	3 (1.9)	2 (2.5)	1 (1.2)	2 (3.0)	4 (4.0)
Major stroke or death	10 (2.0)	7 (2.2)	7 (4.4)	0	0	1 (1.5)	2 (2.0)
MI/AP	12 (2.5)	8 (2.5)	5 (3.1)	2 (2.7)	1 (1.2)	3 (4.5)	1 (1.0)
Any TE	43 (8.8)	26 (8.1)	19 (11.9)	5 (6.3)	2 (2.4)	7 (10.4)	10 (10)
Bleeding	51 (10.5)	31 (9.7)	14 (8.8)	7 (8.9)	10 (12.2)	12 (18)	8 (8.0)
ICH	1 (0.2)	1 (0.3)	1 (0.6)	0	0	0	0
Neck haematoma evacuation	39 (8.0)	22 (6.9)	9 (5.6)	5 (6.3)	8 (9.8)	11 (16.4)	6 (6.0)
Prolonged haemostasis	11 (2.3)	8 (2.5)	4 (2.5)	2 (2.5)	2 (2.4)	1 (1.5)	2 (2.0)

*Note*: The combined outcome parameters stroke and/or death (stroke/death) and major stroke and/or death (major stroke/death) consisted of the patients who had a (major) stroke and/or died during the 30‐day follow‐up.

Abbreviations: AFX, amaurosis fugax; AP, angina pectoris; CEA, carotid endarterectomy; ICH, intracerebral haemorrhage; MI, myocardial infarction; mRS, modified Rankin Scale; RAO, retinal artery occlusion; TE, thrombotic event; TIA, transient ischaemic attack.

In univariate analysis of clinical characteristics, left‐sided CEA (*p* = 0.048) and a history of CHD (*p* = 0.005) were associated with an increased risk and AFX as a presenting symptom (*p* = 0.028) with a decreased risk of perioperative iCVEs (Table 1). After adjusting for age, CHD remained a significant predictor of both iCVEs (age‐adjusted hazard ratio [HR] 2.44, 95% confidence interval [CI] 1.18–5.05) and any thromboembolic event (age‐adjusted HR 2.72, 95% CI 1.45–5.10). Furthermore, both high systolic (age‐adjusted HR 1.01, 95% CI 1.00–1.03) and diastolic blood pressure (age‐adjusted HR 1.02, 95% CI 1.00–1.05) were associated with increased risk of iCVEs.

Several radiological parameters (Table 4) were associated with an increased risk of iCVEs: chronic or covert ipsilateral infarct (age‐adjusted HR 2.27, 95% CI 1.09–4.76), severe leukoaraiosis (age‐adjusted HR 3.36, 95% CI 1.04–10.88), moderate ipsilateral stenosis (age‐adjusted HR 2.14, 95% CI 1.08–4.23) and severe contralateral stenosis (age‐adjusted HR 3.69, 95% CI 1.18–11.5). Severe leukoaraiosis (age‐adjusted HR 3.22, 95% CI 1.53–6.77) and severe contralateral stenosis (age‐adjusted HR 2.98, 95% CI 1.31–6.78) were also associated with an increased risk of any thromboembolic event.

**TABLE 4:** Radiological findings in the carotid stenosis patients with and without any perioperative ischaemic cerebrovascular event (AFX, TIA, stroke, symptom worsening).

Variable	All CEA patients *n* = 488	Patients without iCVE *n* = 455 (93%)	Patients with iCVE *n* = 33 (7%)	Univariate *p* *	Age‐adjusted HR for iCVE	Age‐adjusted HR for any TE
Acute ipsilateral infarct	89 (20.2)	81 (19.7)	8 (27.6)	0.357	1.35 (0.51–3.59)	1.12 (0.47–2.67)
Chronic or covert ipsilateral infarct	107 (24.3)	95 (23.1)	12 (41.4)	0.041	2.27 (1.09–4.76)	1.25 (0.36–4.31)
Chronic ipsilateral infarct	28 (6.3)	24 (5.8)	4 (13.8)	0.10	2.43 (0.85–6.98)	1.67 (0.60–4.71)
Covert ipsilateral infarct	79 (17.9)	71 (17.2)	8 (27.6)	0.21	1.79 (0.79–4.05)	1.36 (0.35–5.29)
Any leukoaraiosis	123 (27.9)	13 (44.8)	110 (26.7)	0.052	1.41 (0.46–4.25)	1.30 (0.54–3.12)
Severe leukoaraiosis	40 (9.1)	32 (7.8)	8 (27.6)	0.002	3.36 (1.04–10.88)	3.22 (1.53–6.77)
Ipsilateral stenosis degree						
50%–69%	165 (33.8)	148 (32.5)	17 (51.5)	0.035	2.14 (1.08–4.23)	1.71 (0.93–3.14)
70%–99%	155 (31.8)	147 (32.3)	8 (24.2)	0.439	1.27 (0.44–3.72)	0.47 (0.06–3.94)
Ipsilateral near‐occlusion (1 mm)	146 (30.0)	139 (30.5)	7 (21.2)	0.326	0.63 (0.27–1.44)	0.34 (0.04–2.91)
Severe contralateral stenosis	34 (7.0)	28 (6.2)	6 (18.2)	0.020	3.69 (1.18–11.5)	2.98 (1.31–6.78)
Contralateral occlusion	32 (6.6)	30 (6.6)	2 (6.1)	1.000	0.64 (0.08–5.26)	1.67 (0.59–4.74)
Plaque ulceration	94 (21.0)	89 (21.2)	5 (17.2)	0.814	1.05 (0.35–3.09)	0.91 (0.38–2.15)
Circulus Willis anomaly						
A1 hypoplasia	27 (5.5)	27 (5.9)	0	0.243	ND	ND
Ipsilateral fetal PCA	18 (3.7)	18 (4.0)	0	0.392	ND	ND
A1 hypoplasia and ipsilateral fetal PCA	1 (0.2)	1 (0.2)	0	1.000	ND	ND

Abbreviations: A1, A1 segment of the anterior cerebral artery; AFX, amaurosis fugax; CEA, carotid endarterectomy; HR, hazard ratio; iCVE, ischaemic cerebrovascular event; PCA, posterior cerebral artery; TIA, transient ischaemic attack. Available from 481 to 487 patients.

* *p* < 0.05 is considered statistically significant.

None of the potential biomarkers was associated with the risk of iCVEs (Table 2). High‐sensitivity C‐reactive protein was associated with an increased risk of any thromboembolic event (age‐adjusted HR 1.01, 95% CI 1.00–1.03).

In a subgroup analysis by the onset of iCVE (within 6 h, during hospital stay or within 30 days after CEA), severe leukoaraiosis, covert or chronic infarct, moderate ipsilateral stenosis and stroke as a presenting symptom were significantly associated with iCVEs within 6 h of CEA, and CHD and severe contralateral stenosis with iCVE during hospital stay. A detailed description of the analysed variables is shown in Table S1.

In a sensitivity analysis, only patients who suffered a perioperative ischaemic stroke (*n* = 21) were analysed, thus excluding patients who had TIA or symptom worsening. The age‐adjusted hazard ratios for the history of CHD (HR 4.81, 95% CI 1.82–12.72), chronic or covert ipsilateral infarct in brain imaging (HR 3.59, 95% CI 1.46–8.85) and severe leukoaraiosis (HR 4.10, 95% CI 1.37–12.26) remained significant. In addition, stroke as the presenting symptom (HR 2.98, 95% CI 1.26–7.04) was a significant predictor for a perioperative ischaemic stroke.

Finally, the association between iCVE‐free survival time and potential predictor variables was investigated, using Cox proportional hazards regression modelling (Table 5). It was found that female gender (HR 3.03, 95% CI 1.30–7.04), a history of CHD (HR 3.59, 95% CI 1.52–8.47), chronic or covert ipsilateral infarct in brain imaging (HR 2.32, 95% CI 1.01–5.34) and severe leukoaraiosis (HR 2.63, 95% CI 1.07–6.47) were the best independent predictors of perioperative iCVE (Table 5).

**TABLE 5:** Cox proportional hazards regression models for perioperative stroke, TIA or symptom worsening in carotid stenosis patients undergoing carotid endarterectomy.

Variable	Model A			Model B		
	*p* value	HR	95% CI	*p* value	HR	95% CI
Age	0.676	0.99	0.94–1.04	n.i.	n.i.	n.i.
Female gender	0.073	2.09	0.93–4.67	0.010	3.03	1.30–7.04
Left‐sided CEA	0.249	1.57	0.73–3.38	0.072	2.17	0.93–5.02
CHD	0.005	3.22	1.42–7.30	0.004	3.59	1.52–8.47
Presenting symptom AFX	0.122	0.20	0.03–1.53	n.i.	n.i.	n.i.
Chronic or covert ipsilateral infarct	0.065	2.05	0.96–4.41	0.049	2.32	1.01–5.34
Severe leukoaraiosis	0.016	2.95	1.22–7.11	0.035	2.63	1.07–6.47
Moderate ipsilateral stenosis	0.354	1.43	0.67	n.i.	n.i.	n.i.
Severe contralateral stenosis	0.140	2.26	0.77–6.65	n.i.	n.i.	n.i.

*Note*: Model A includes age, sex and the variables significant by univariate analysis in this study. Model B includes age, sex and the variables showing association with a *p* value below 0.20 in the univariate analysis but subjected to backward elimination.

Abbreviations: AFX, amaurosis fugax; CEA, carotid endarterectomy; CHD, coronary heart disease; CI, confidence interval; HR, adjusted hazard ratio; n.i., not included; TIA, transient ischaemic attack.

## DISCUSSION

In this study, predictors for perioperative iCVE in CS patients within 30 days after CEA were investigated, emphasizing cerebrovascular imaging and potential stroke biomarkers. Imaging markers of ischaemic cerebrovascular disease, severe leukoaraiosis and covert or chronic ipsilateral infarct in brain imaging, as well as moderate ipsilateral stenosis were significantly associated with the risk of perioperative iCVEs in our cohort. Furthermore, several earlier reported predictors [7, 19, 27, 28] could be replicated including the type of presenting event (AFX with decreased risk), female gender, a history of CHD, left‐sided CEA, high preoperative blood pressure and severe contralateral stenosis [19, 20, 21, 24, 27]. In contrast, none of the earlier reported blood biomarkers was significantly associated with iCVEs.

Cerebral small vessel disease, manifesting radiologically as subcortical white matter lesions, typically results from underlying vascular risk factors, most commonly hypertension, smoking and diabetes, which damage the small arterioles in the subcortical white matter, leading to endothelial dysfunction. Small vessel disease is associated with lacunar stroke and unfavourable outcomes after ischaemic stroke in the acute phase, worsening long‐term outcomes and cognitive disturbances [29]. It was also linked to an increased 30‐day risk of stroke after CEA in a substudy of NASCET [30] and after carotid artery stenting, but not after CEA in the International Carotid Stenting Study [12]. Patients with severe cerebral small vessel disease have a reduced cerebral blood flow and impaired cerebral autoregulation which may render them more susceptible to ischaemia. A recent study showed a U‐shaped relationship between diastolic blood pressure and outcome after acute ischaemic stroke in cerebral small vessel disease patients [31]. Hence these patients may be especially vulnerable to compromised cerebral perfusion and rapid blood pressure changes during surgery or the postoperative time period and may benefit from intensive follow‐up and optimization of blood pressure level in the prevention of perioperative iCVEs. Most of the iCVEs in our study occurred within 6 h of surgery, indicating increased vulnerability to blood pressure fluctuations and highlighting the critical need for careful blood pressure management in this group of patients.

The authors are not aware of previous studies reporting an association between chronic or covert infarcts and the risk of perioperative stroke, although covert ipsilateral infarcts have been associated with the risk of stroke in asymptomatic CS patients on long‐term follow‐up [1]. In our study, most of the infarcts in patients with perioperative iCVE were covert (Table 4) and non‐lacunar infarcts located subcortically in the ipsilateral anterior circulation or on watershed areas. Due to the small number of lesions and their scattered distribution, drawing firm conclusions on the stroke mechanisms is challenging, but generalized atherosclerosis and earlier thromboembolic events from ipsilateral carotid plaques are the most likely explanations.

Moderate ipsilateral CS has not been associated with the risk of perioperative stroke after CEA before. In our study these patients had more hypercholesterolaemia but otherwise they did not differ from the other patients regarding the burden of vascular risk factors. Earlier studies have shown that those with moderate CS usually have soft unstable plaques whereas severe CS more often are calcified plaques [32]. The soft plaques are more prone to cause symptoms by plaque rupture or erosion. This has been well shown in patients with coronary artery disease who have suddenly died of cardiac infarction [33]. It is hypothesized that moderate stenoses are more often lipid‐rich, inflamed soft plaques which are prone to rupture and thromboembolism whilst manipulated during the surgical procedure.

In our study severe contralateral stenosis was associated with more perioperative iCVEs. One earlier study, the New York Carotid Artery Surgery study, showed that individuals with stenosis ranging from 50% to 99% in the contralateral internal carotid exhibited a 44% higher risk‐adjusted rate of complications, probably attributable to reduced capacity for collateral blood flow [34]. On the other hand, contralateral occlusion has been associated with 30‐day stroke/death, any in‐hospital stroke, in‐hospital ipsilateral and contralateral stroke, and prolonged hospital durations after CEA [30]. The existence of sufficient compensatory collateral flow has the potential to shield patients from the worsening of clinical symptoms [35]. Collateral circulation through the circle of Willis and secondary networks, such as the ophthalmic artery and leptomeningeal vessels, influences stroke risk in patients with severe CS [36]. In our study, it was found that circle of Willis variants alone were not associated with the risk of iCVEs. This is probably due to the complexity of collateral recruitment and the haemodynamic factors involved, which would require advanced neurovascular imaging to be fully unravelled. In our cohort the patients who had severe contralateral stenosis had a higher burden of comorbidities; they had more coronary artery disease, major cardiovascular event, diabetes mellitus and probable metabolic syndrome, which can all contribute to the increased risk of iCVEs perioperatively.

In our study, asymptomatic patients showed more perioperative iCVEs (10%) than previously shown in other studies. This may have resulted from patient selection, since in our treatment centre CEA is considered in those asymptomatic patients who are at high risk of stroke long term, for example due to high loading of vascular risk factors or multiple stenoses of the cervical arteries. In our cohort, 50% of all the asymptomatic patients suffered from severe (>69%) contralateral stenosis, rendering them a high perioperative risk. As stated in the Stroke Prevention Potential study [37], it is more crucial to choose the right patient for surgery and perform surgery at the right time than only look at the stroke incidence. In our centre, the long‐term risk of perioperative strokes after CEA is less than 3% in preoperatively asymptomatic patients.

Current treatment options, including DAPT, IVT and endovascular thrombectomy (EVT), shape the implications of our study on CS and perioperative complication risks. Our findings highlight the challenge of balancing DAPT's stroke prevention benefits against the increased haemorrhagic risk in patients with small vessel disease, a question that future research needs to clarify. IVT has remained stable in Finland over the past 10–15 years, although the risk of ICH related to IVT is notably higher in patients with small vessel disease [38]. Another unresolved question is the optimal acute treatment for patients with CS and minor symptoms. According to the recent TEMPO trial [39], IVT did not show a benefit and may even pose a risk in patients with minor strokes, although a subgroup analysis for patients with CS was not conducted. EVT, although revolutionary, applies to a limited subset of stroke patients, with only a small percentage being CS cases, reducing its relevance to the broader CS population.

In conclusion, female gender, history of CHD, severe small vessel disease and chronic or covert ipsilateral infarcts in brain imaging were the most important predictors for the risk of a perioperative iCVE in our cohort. Earlier studies have concentrated on patient demographics, comorbidities or surgery‐related technical factors, but our study shows that evaluation of the frailties of cerebrovascular circulation from the brain and vessel imaging is as important since the resilience of the cerebral tissue to ischaemia is highly relevant and should not be forgotten.

## LIMITATIONS

Our study has some limitations. The HeCES2 study enrolled all patients scheduled for CEA in a large tertiary hospital in the years 2012–2015. Although the study involved a sizeable patient cohort, it may lack statistical power to detect certain associations observed in larger clinical trials. Furthermore, due to a low incidence of iCVEs, the subgroup analysis needs to be interpreted with caution. Although the recruitment for this study was consecutive, a small fraction (5%) of patients scheduled for CEA had not been recruited. However, these recruitment gaps occurred sporadically during the summer breaks when staffing constraints impeded recruitment efforts, and accordingly these gaps were random rather than systematic.

## AUTHOR CONTRIBUTIONS

**Henrietta Törmänen:** Conceptualization; writing – original draft; methodology; formal analysis; data curation. **Suvi Koskinen:** Writing – review and editing; data curation. **Krista Nuotio:** Writing – review and editing; data curation. **Pirkka Vikatmaa:** Writing – review and editing; data curation. **Petri T. Kovanen:** Writing – review and editing. **Lauri Soinne:** Writing – review and editing; data curation. **Perttu J. Lindsberg:** Data curation; supervision; writing – review and editing; methodology. **Petra Ijäs:** Supervision; conceptualization; writing – review and editing; formal analysis; data curation; methodology.

## FUNDING INFORMATION

Aarne Koskelo Foundation and the Hospital District of Helsinki and Uusimaa Research Grants.

## CONFLICT OF INTEREST STATEMENT

The authors declare no conflict of interest.

## Supporting information


Data S1.

## Data Availability

The data are available upon author request so that anonymity is not compromised.
