# Supplementary material for: Carotid endarterectomy and the risk of perioperative stroke: The importance of chronic ischaemic lesions and small vessel disease
Source: Eur J Neurol. 2025 Jan 3;32(1):e16551. doi: 10.1111/ene.16551 (PMC11696523; doi:10.1111/ene.16551)
Supplement: Supplementary file 1 — Data S1. [file ENE-32-e16551-s001.docx]

**Carotid endarterectomy and the risk of perioperative stroke:** **the importance of chronic ischemic lesions and small vessel disease**

**^1,2^Henrietta Törmänen, ^2,3^Suvi Koskinen, ^1,2^Krista Nuotio, ^4^Pirkka Vikatmaa, ^5^Petri T. Kovanen, ^1^Lauri Soinne, ^1,2^Perttu J. Lindsberg, ^1,2^Petra Ijäs.**

**Supplemental Methods.**

**Table 1. Baseline characteristics in operated carotid stenosis patients with and without any perioperative ischemic cerebrovascular events (AFX, TIA, stroke, symptom worsening) by the onset of ischemic cerebrovascular event.**

|  | **Patients without iCVE N=455** | **iCVE within 6 hours of CEA** | **p-value** | **iCVE during hospital stay** | **p-value** | **iCVE after hospitalization, within 30 days** | **p-value** |
| --- | --- | --- | --- | --- | --- | --- | --- |
| Gender (female) | 145 | 5 (31) | 1.000 | 7 (50) | 0.159 | 1 (33) | 1.000 |
| Age (mean/SD) | 69.6±8.4 | 72 ±10 | 0.258 | 72 ±9.2 | 0.271 | 66±4.6 | 0.458 |
| smoking | 168 (37) | 3 (19) | 0.188 | 4 (29) | 0.588 | 3 (100) | 0.051 |
| Heavy alcohol consumption | 50 (11.0) | 1 (6.3) | 1.000 | 0 | 0.381 | 1 (33) | 0.299 |
| ***Type of presenting symptom*** |  |  |  |  |  |  |  |
| stroke | 122 (27) | 9 (56) | **0.019** | 5 (36) | 0.541 | 0 | 0.568 |
| RAO | 23 (5.1) | 0 | 1.000 | 1 (7.1) | 0.526 | 0 | 1.000 |
| TIA | 76 (17) | 0 | 0.087 | 2 (14) | 1.000 | 1 (33) | 0.425 |
| AFX | 81 (18) | 1 | 0.327 | 0 | 0.143 | 0 | 1.000 |
| Asymptomatic | 90 (20) | 4 (25) | 0.537 | 5 (83) | 0.403 | 1 | 1.000 |
| Suspect | 63 (13) | 2 (13) | 1.000 | 1 (17) | 0.403 | 1 | 1.000 |
| ***Comorbidities*** |  |  |  |  |  |  |  |
| Hypertension | 369 (81) | 8 (50) | 0.450 | 13 (93) | 0.484 | 3 (100) | 1.000 |
| Diabetes | 151 (33) | 6 (38) | 0.789 | 4 (29) | 1.000 | 2 (67) | 0.260 |
| Hypercholesterolemia | 414  (91.0) | 15 (94) | 1.000 | 14 (100) | 1.000 | 3 (100) | 1.000 |
| AF | 76 (17) | 4 (25) | 0.329 | 4 (29) | 0.273 | 0 | 1.000 |
| CHD | 161 (35) | 7 (44) | 0.597 | 11 (79) | **0.001** | 2 (67) | 0.289 |
| Probable metabolic syndrome | 184 (40) | 8 (50) | 0.450 | 8 (57) | 0.271 | 1 (33) | 1.000 |
| ***Radiological parametres*** |  |  |  |  |  |  |  |
| Any leukoaraiosis | 110 (24) | 7 (50) | 0.068 | 6 (50) | 0.098 | 0 | 0.569 |
| severe leukoararaiosis | 32 (7) | 4(29) | **0.023** | 4 (33) | 0.487 | 0 | 1.000 |
| covert or chronic infarction | 95 (21) | 7 (50) | **0.049** | 4 (33) | 0.487 | 1(33) | 0.547 |
| moderate ipsilateral stenosis | 148 (33) | 10 (63) | **0.027** | 7 (50) | 0.246 | 0 | 0.554 |
| severe contralateral  stenosis | 28 (6.2) | 2 (13) | 0.271 | 4 (29) | **0.011** | 0 | 1.000 |
| ***Medication*** |  |  |  |  |  |  |  |
| SAPT | 236 (52) | 8 (50) | 1.000 | 6 (43) | 0.592 | 1 (33) | 0.611 |
| DAPT | 20 (4.4) | 1 (6.3) | 0.524 | 1 (7.1) | 0.478 | 0 | 1.000 |
| LMWH | 202 (44) | 8 (50) | 0.799 | 9 (64) | 0.176 | 2 (67) | 0.588 |
| Any recurrent event prior to CEA | 43 (9.5) | 1 (6.3) | 0.588 | 0 | 0.628 | 0 | 1.000 |
| SBP at arrival | 152±26.9 | 156±21 | 0.606 | 166 ±23 | 0.105 | 141±22 | 0.541 |
| DBP at arrival | 78 ± 14.9 | 78±16 | 0.989 | 85 ±20 | 10.179 | 66±12 | 0.591 |
| IVT | 22 (4.8) | 1 (2.3) | 0.557 | 0 | 1.000 | 0 | 1.000 |
| Perioperative bleeding | 47 (10) | 2 (13) | 0.678 | 2 (14) | 0.649 | 0 | 1.000 |

Data are presented as n (%), or mean ± standard deviation.^*^<.05 is considered statistically significant. Chi-square or Fisher’s exact test for dichotomous or categorical variables, Mann-Whitney test for ordinal variables and Student’s t-test for continuous variables were used. Patients who still smoked for as long as 1 year before the index symptom were classified as smokers. Heavy alcohol consumption in women was rated as more than 12–16 doses (one dosage of alcohol is 12 g of pure ethanol) and in men more than 23–24 doses of alcohol per week. iCVE= ischemic cerebrovascular event, CHD= coronary heart disease, DM = diabetes mellitus, RAO = retinal artery occlusion, TIA = transient ischemic attack, AFX = amaurosis fugax, CEA=carotid endarterectomy, AF=atrial fibrillation, DAPT= dual antiplatelet treatment, SAPT= single antiplatelet therapy, LMWH= low-molecular weight heparin, SBP=systolic blood pressure, DBP=diastolic blood pressure.
